# Influences of Experience of Violence and Cognitive-Emotion Regulation Strategies on Psychiatric Nurses’ Post-Traumatic Stress

**DOI:** 10.3390/healthcare13233090

**Published:** 2025-11-27

**Authors:** Hyun Jae Park, Seung Hyun Hong, Nam Hee Kim, Sung Hee Shin

**Affiliations:** 1 Department of Nursing, Graduate School, Kyung Hee University, Seoul 02447, Republic of Korea; starzzz93@khu.ac.kr (H.J.P.); hshsky@khu.ac.kr (S.H.H.); nhk0916@korea.kr (N.H.K.); 2 College of Nursing Science, Kyung Hee University, Seoul 02447, Republic of Korea

**Keywords:** psychiatric nurses, violence experience, cognitive-emotion regulation strategies, post-traumatic stress

## Abstract

**Highlights:**

**What are the main findings?**

- Psychiatric nurses experiencing workplace violence showed high post-traumatic stress levels, exceeding clinical cutoff thresholds, with verbal violence being the most frequently experienced type.
- Maladaptive cognitive-emotion regulation strategies were the strongest predictor of post-traumatic stress, followed by violence experiences and educational level, explaining 62.6% of variance.

**What are the implications of the main findings?**

- Healthcare institutions should implement comprehensive violence prevention programs and provide immediate psychological support for all types of workplace violence, including verbal abuse, which is often underreported.
- Interventions should focus on teaching psychiatric nurses to reduce maladaptive cognitive-emotion regulation strategies while enhancing adaptive coping mechanisms to mitigate post-traumatic stress development.

**Abstract:**

**Background/Objectives**: Psychiatric nurses are constantly exposed to physical and verbal violence from patients with mental illnesses, which can lead to post-traumatic stress (PTS). This study investigated the correlations among psychiatric nurses’ experiences of violence, cognitive-emotion regulation strategies, and PTS and sought to identify factors associated with PTS. Although workplace violence and its psychological effects have been examined among nurses, little is known about how cognitive-emotion regulation influences PTS in psychiatric nurses who are frequently exposed to violence. Understanding these mechanisms is crucial for developing interventions to support their mental health. **Methods**: This was a cross-sectional, descriptive correlational study. Participants were 140 psychiatric nurses with more than one year of clinical experience working in psychiatric wards at university, general, and psychiatric hospitals in South Korea. Collected data were analyzed using SPSS/WIN 25.0. A hierarchical regression analysis was performed to identify factors influencing the nurses’ PTS. Hierarchical regression analysis was performed in three steps: demographic variables were entered first, followed by violence experience, and finally cognitive-emotion regulation strategies. All assumptions of linearity, normality, and homoscedasticity were satisfied. **Results**: In Model 3, after controlling for demographic and work-related variables, maladaptive emotion regulation strategies, experiences of violence, and education level emerged as significant predictors of PTS among psychiatric nurses. These variables together explained a substantial proportion of the variance in PTS. **Conclusions**: To reduce PTS among psychiatric nurses, it is necessary to develop and implement violence prevention and coping programs, stress and mental health management initiatives, and educational programs. Based on the findings, hospitals should strengthen organizational systems by establishing structured mechanisms for reporting and debriefing after violent incidents. In addition, hospitals should provide regular training on cognitive-emotion regulation and enhance institutional support to help nurses manage the psychological impact of workplace violence. Such interventions may not only minimize violent incidents but also reduce reliance on maladaptive cognitive-emotion regulation strategies. While the findings provide important insight, the cross-sectional design limits causal inference. Further longitudinal research is recommended to verify these relationships.

## 1. Introduction

Psychiatric nurses frequently encounter verbal and physical aggression in clinical settings, which can negatively affect their psychological health and work functioning [1]. Their vulnerability is heightened by patient-related symptoms—such as hallucinations or delusions—and by environmental factors inherent to closed psychiatric wards [2]. Prior reviews consistently show that verbal violence is the most common form experienced by psychiatric nurses, followed by emotional and physical violence [3]. Exposure to verbal, emotional, or physical aggression in the workplace can function as a traumatic event that disrupts psychiatric nurses’ psychological stability and evokes PTS symptoms [4]. Prolonged or repeated exposure may further increase the risk of PTSD and negatively affect clinical performance, job satisfaction, and turnover intention through both direct psychological effects and indirect impacts on work performance [5,6]. People may differ substantially in their psychological responses to traumatic events, and only a small proportion of individuals develop PTSD despite widespread exposure to trauma [7]. This suggests that certain protective characteristics help maintain psychological stability and reduce the likelihood of developing PTSD [8]. Even under similar stressors, some individuals adapt more effectively, which may be influenced by how well they use cognitive coping mechanisms—particularly cognitive-emotion regulation strategies—to manage distressing emotions in challenging situations [9]. Emotion regulation plays a critical role in sustaining well-being and functional adaptation by reducing stress associated with negative emotions and preventing maladaptive responses [10]. Cognitive-emotion regulation strategies refer to the cognitive processes individuals use to interpret and manage stress-related information [11]. Garnefski et al. [11] classify these strategies into more adaptive and less adaptive forms, and Kim [12] adapted this framework for the Korean context, distinguishing adaptive and maladaptive cognitive-emotion regulation strategies.

Workplace violence experienced by psychiatric nurses is strongly associated with PTS and can undermine both personal well-being and the quality of patient care. Prior studies show that exposure to severe violence is one of the strongest predictors of PTSD among psychiatric nurses [5], and a meta-analysis reported that nurses with violence exposure were more than twice as likely to develop PTS compared to those without such experiences [13]. In addition, maladaptive cognitive-emotion regulation strategies have been identified as partial mediators in the relationship between violence exposure and PTS [14], supporting the proposition that individual regulatory patterns contribute to differences in trauma outcomes. Consistent with this, Jang and Shin found that aggressive incidents were negatively associated with adaptive cognitive-emotion regulation strategies among mental health professionals [15]. Collectively, these findings indicate that violence in psychiatric settings can function as a significant traumatic stressor that affects nurses’ mental health, patient safety, and overall clinical functioning.

Despite this evidence, model-based investigations examining how workplace violence and cognitive-emotion regulation strategies jointly influence PTS among psychiatric nurses remain limited. Most previous research has examined only direct associations between violence and PTS without testing integrated models incorporating mediating or moderating effects [5]. Furthermore, many studies have focused on general or emergency department nurses rather than psychiatric nurses [13,16,17,18], leaving the unique dynamics of locked and semi-locked psychiatric units underrepresented. Although cognitive-emotion regulation instruments have been used in Korean nursing research [12,19], few studies have empirically evaluated the combined relationships among violence exposure, emotion regulation strategies, and PTS specifically within psychiatric nursing populations. These gaps highlight the need for empirical model testing in this specialized context.

Therefore, this study examines how workplace violence and cognitive-emotion regulation strategies are associated with PTS among psychiatric nurses. The findings are expected to provide empirical evidence for developing interventions to reduce trauma-related stress and support healthy emotion regulation in this population.

## 2. Materials and Methods

### 2.1. Research Design

This descriptive, cross-sectional study examines the correlation between psychiatric nurses’ experiences of violence, cognitive-emotion regulation strategies, and PTS, and identifies factors influencing PTS. This cross-sectional design was selected as appropriate for examining the relationships among variables and identifying factors associated with PTS at a specific point in time. While we acknowledge that PTSD symptoms may evolve over time, this design allows us to identify current correlations and risk factors that can inform the development of immediate intervention programs. Longitudinal studies would be valuable for future research to examine the temporal evolution of these relationships.

### 2.2. Participants

The participants in this study were 140 psychiatric nurses with at least one year of clinical experience in psychiatry, working at university, general, and psychiatric hospitals in South Korea. This criterion was based on previous studies [20,21] which found that one year after clinical training, nurses’ knowledge and skills stabilize, and their job performance becomes relatively stable.

Sample size was calculated using G*Power 3.1.9. For the multiple regression analysis, the following parameters were used: a medium effect size (f^2^ = 0.15), a significance level of 0.05, power of 0.80, and 14 independent variables (11 general characteristics, experience of violence, adaptive cognitive-emotion regulation strategies, and maladaptive cognitive-emotion regulation strategies). The medium effect size was selected based on several considerations: (1) Cohen’s conventional guideline of f^2^ = 0.15 for medium effects in multiple regression, (2) effect sizes reported in previous studies examining predictors of PTS in nursing populations, which ranged from 0.12 to 0.35 [13], and (3) the exploratory nature of including multiple demographic covariates alongside theoretically driven predictors, which suggested a moderate effect estimate was appropriate. However, although the required sample size was met, including 14 independent variables in a model with a relatively modest sample (N = 140) may increase the risk of overfitting. These predictors were selected because they reflect variables consistently identified as relevant to PTS in previous studies; nevertheless, this potential limitation should be considered when interpreting the regression results.

### 2.3. Instruments

The instruments used in this study consisted of an 84-item structured self-report questionnaire covering: 12 items on general participant characteristics, 16 items on experiences of violence, 36 items on cognitive-emotion regulation strategies, and 20 items on PTS. Permission to use each instrument was obtained from its respective original authors, translators, and developers prior to administration.

While all instruments used in this study have been translated or adapted for Korean populations and previously validated, cultural and contextual factors specific to psychiatric nursing in Korea may still influence responses. In addition, because all measures were self-reported and collected at a single time point, the possibility of common method bias cannot be fully ruled out.

#### 2.3.1. Violence Experiences

Experiences of violence were measured using a tool developed by Son [16] and later modified and refined for nurses by Yoon [17]. The instrument consists of 16 items: four addressing verbal violence (based on weekly occurrences), five addressing physical threats (monthly occurrences), and seven addressing physical violence (annual occurrences). It uses a 5-point Likert scale (0–4), with total scores ranging from 0 to 64. Higher scores indicate a greater experience with each type of violence. For this study, the instrument’s reliability was indicated by a Cronbach’s α of 0.87.

#### 2.3.2. Cognitive-Emotion Regulation Strategies

Cognitive-emotion regulation strategies were assessed using the Cognitive-Emotion Regulation Questionnaire, developed by Garnefski et al. [11] and translated into Korean by Kim [12]. This instrument consists of 36 items: 20 items on adaptive emotion regulation strategies (Positive Refocusing, Positive Reappraisal, Putting into Perspective, Refocus on Planning, and Acceptance) and 16 items on maladaptive emotion regulation strategies (Rumination, Self-Blame, Blaming Others, and Catastrophizing). Responses were rated on a 5-point Likert scale ranging from “not at all” (1) to “very much” (5). The score range for adaptive strategies is 20–100 and 16–80 for maladaptive strategies, with higher scores indicating greater use of the corresponding strategy. In Kim’s [12] study, Cronbach’s α ranged from 0.66 to 0.85 for adaptive strategies and from 0.53 to 0.78 for maladaptive strategies. In the present study, Cronbach’s α was 0.92 for adaptive strategies and 0.85 for maladaptive strategies.

#### 2.3.3. Post-Traumatic Stress

PTS was measured using the Korean version of the PTSD Checklist for DSM-5 (PCL-5), which was developed by Blevins et al. [22] and translated and validated into Korean by Kim et al. [23]. This 20-item tool assesses PTSD symptoms according to DSM-5 criteria. Responses were rated on a 5-point Likert scale ranging from “Not at all” (0 points) to “Extremely” (4 points), for a total score range of 0–80. Higher scores indicate greater PTS symptom severity. A cutoff score of 31–33 is generally recommended for the PCL-5 [24]. The tool’s reliability was Cronbach’s α = 0.97 in the study by Kim et al. [23] and Cronbach’s α = 0.96 in the present study.

### 2.4. Data Collection and Ethical Considerations

Prior to data collection, approval was obtained from the K University Institutional Review Board (KHSIRB-23-428). The data collection period was from 16–31 October 2023, and data were gathered using convenience sampling to facilitate recruitment. An online Naver community forum used exclusively by psychiatric nurses was utilized. After explaining the purpose and intent of this study to the forum administrator and requesting their cooperation, a Google survey link was posted. Additionally, the first question on the Google Form survey confirmed eligibility by asking if the respondent had been working in a psychiatric ward for more than one year; if eligibility criteria were not met, the survey was terminated. Although online self-administered surveys may introduce response bias due to the lack of supervision, several procedures were implemented to enhance data quality. To prevent duplicate submissions, each user ID was permitted to respond to the survey only once. To protect participants, this study’s purpose and intent were explained via the Google Form before participation. To ensure emotional protection of participants, the informed consent statement included information about the potentially sensitive nature of the questions regarding violence experiences and psychological symptoms. Participants were informed they could skip questions or withdraw at any time without penalty. Contact information for mental health support resources, including employee assistance programs and crisis hotlines, was provided at the end of the survey for participants who might experience distress. All data were anonymized to maintain participant anonymity and confidentiality, and a temporary code was used for identification. The computer and removable drive where the collected data were stored were password-protected to prevent access by anyone other than the researchers. A total of 140 psychiatric nurses completed the online survey and were included in the final analysis. The survey link was distributed through a closed online community for psychiatric nurses, and only individuals who met the eligibility criterion of having at least one year of psychiatric nursing experience were able to proceed to the questionnaire. All 140 respondents met the eligibility requirement and provided complete responses; therefore, no cases were excluded due to ineligibility or missing data.

### 2.5. Data Analysis

Data from this study were analyzed using IBM SPSS Statistics for Windows, Version 25.0 (IBM Corp., Armonk, NY, USA). Although online self-administered surveys may introduce response bias due to the lack of supervision, several procedures were implemented to enhance data quality. All collected surveys were reviewed for completeness. As the online survey required responses to all items before submission, there were no missing data in the final dataset. All 140 completed surveys were included in the analysis. The general characteristics of the participants were analyzed using frequencies, percentages, means, and standard deviations. Descriptive statistics, including means, standard deviations, skewness, and kurtosis, were used to analyze participants’ experiences with violence, cognitive-emotion regulation strategies, and PTS levels. Independent t-tests and one-way ANOVAs were conducted to analyze differences in PTS based on participant characteristics, with post hoc comparisons performed using Scheffe’s test. Pearson’s correlation coefficients were calculated to examine the relationships among participants’ experiences of violence, cognitive-emotion regulation strategies, and PTS. A hierarchical regression analysis was performed to examine the effects of participant characteristics, experiences of violence, and adaptive and maladaptive emotion regulation on PTS. The reliability of the instrument was assessed using Cronbach’s alpha (α). In addition, because multiple bivariate analyses were conducted, *p*-values were presented without adjustment, and the results should be interpreted in light of the increased risk of Type I error. Due to the exploratory nature of these bivariate analyses and to avoid overly conservative Type I error control that might exclude relevant covariates, we present unadjusted *p*-values. Findings should be interpreted considering the increased possibility of false-positive results when conducting multiple comparisons.

## 3. Results

### 3.1. General Characteristics of Participants and Differences in PTS According to General Characteristics

The general characteristics of the participants were assessed, including gender, age, religion, level of education, marital status, hospital type, job title, certification status, total clinical experience, total psychiatric clinical experience, frequency of emergency response training, and the measures deemed necessary for post-incident care at the ward or hospital level (Table 1).

Regarding gender, the majority of participants were female (92.9%). The mean age was 30.76 years, with those between 30 and 35 years old forming the largest group (42.1%). In terms of religion, 63.6% of participants reported having a religious affiliation. As for education, the largest proportion of participants held a university-level degree (87.1%). In terms of marital status, single participants (79.3%) outnumbered those who were married. Regarding hospital type, most participants worked in university hospitals (45.7%). The majority held the position of staff nurse (93.6%). Certifications such as Level 1 or 2 Mental Health Nurse or Mental Health Specialist Nurse were held by 67.1% of participants. The average total clinical experience was 5.67 years, with participants having 5–8 years of experience constituting the largest group (35.0%). The average psychiatric clinical experience was 3.28 years, with the majority having less than 3 years of experience in the field (55.0%). Concerning emergency response training, most participants had undergone fewer than five sessions (75.7%). As for desired measures in response to experiences of violence, participants selected the following in order of preference: patient transfers (67.1%), changes in work assignments such as emergency leave (64.3%), counseling services (63.6%), and other measures (3.0%).

Statistically significant differences in PTS levels were observed based on education level, hospital type, and total clinical experience (Table 1). Regarding education, participants with an associate’s degree exhibited the highest PTS levels (44.83 points), followed by those with a bachelor’s degree (38.33 points) and those with a graduate degree (21.17 points), indicating that lower educational attainment was associated with higher PTS levels (F = 6.48, *p* = 0.002). With respect to hospital type, nurses working in psychiatric specialty hospitals reported the highest PTS levels (43.05 points), followed by those in university hospitals (34.75 points) and general hospitals (34.57 points) (F = 3.52, *p* = 0.032). An inverse relationship was found between clinical experience and PTS levels (F = 5.93, *p* = 0.001): nurses with less than three years of experience had the highest scores (48.33 points), followed by those with 5 to less than 8 years (34.51 points), 3 to less than 5 years (33.82 points), and 8 or more years of experience (33.63 points).

No statistically significant differences were observed in PTS levels based on sex (F = −0.35, *p* = 0.729), age (F = 2.60, *p* = 0.078), religion (F = −1.41, *p* = 0.161), marital status (F = 1.70, *p* = 0.091), job title (F = 0.60, *p* = 0.549), certification status (F = −0.61, *p* = 0.543), total psychiatric clinical experience (F = 2.41, *p* = 0.093), or frequency of emergency response training (F = 2.96, *p* = 0.055).

### 3.2. Levels of Violence Experience, Cognitive-Emotion Regulation Strategies, and PTS

The levels of violence experiences, cognitive-emotion regulation strategies, and PTS among psychiatric nurses are presented in Table 2. The mean score for experiences of violence was 28.60 (±13.13) out of a maximum of 64. Among the subfactors, the mean score for verbal violence was 9.01 (±3.43), for physical threats was 9.68 (±4.67), and for physical violence was 9.91 (±6.70). When calculated as mean per-item scores on a 4-point scale, verbal violence scored 2.25 (±0.86), physical threats scored 1.94 (±0.93), and physical violence scored 1.42 (±0.96), indicating that verbal violence had the highest severity. The mean PTS score was 37.14 (±17.28) out of a total of 80. Adaptive cognitive-emotion regulation strategies had a mean score of 65.77 (±13.29) out of 100, while maladaptive cognitive-emotion regulation strategies had a mean score of 47.18 (±9.75) out of 80. On a 5-point scale, the mean per-item score for adaptive strategies was 3.29 (±0.66), and for maladaptive strategies, it was 2.95 (±0.61). To assess whether the data met the assumption of normality, skewness and kurtosis were examined. In accordance with the standard that the absolute value for skewness must be less than ±2 and for kurtosis less than ±7 [25], all variables met these criteria. This indicates that the data satisfied the assumption of normality, allowing for the use of parametric statistical methods, such as regression analysis, without concern for the data’s distribution.

### 3.3. Correlations Among Psychiatric Nurses’ Violence Experience, Cognitive-Emotion Regulation Strategies, and PTS

The correlations among experiences of violence, adaptive emotional regulation strategies, maladaptive emotion regulation strategies, and PTS in psychiatric nurses are presented in Table 3. A positive correlation was observed between experiences of violence and PTS (r = 0.60, *p* < 0.001). Additionally, experiences of violence showed positive correlations with both adaptive (r = 0.30, *p* < 0.001) and maladaptive (r = 0.48, *p* < 0.001) emotion regulation strategies. While adaptive emotion regulation strategies did not have a significant correlation with PTS (r = 0.16, *p* = 0.056), a positive correlation was found between adaptive emotion regulation strategies and maladaptive emotion regulation strategies (r = 0.26, *p* = 0.002). Furthermore, a strong positive correlation was observed between maladaptive emotion regulation strategies and PTS (r = 0.72, *p* < 0.001).

### 3.4. Factors Influencing PTS in Psychiatric Nurses

A hierarchical regression analysis was performed in three sequential steps to examine the effects of participant characteristics, experiences of violence, and cognitive-emotion regulation strategies on PTS. In Model 1, control variables were entered, including demographic characteristics that showed statistically significant associations with PTS in bivariate analyses (education level [reference = associate degree], hospital type [reference = university hospital], and total clinical experience), as well as variables identified in the literature as potentially influencing PTS (gender [reference = male] and marital status [reference = unmarried]). All control variables except total clinical experience were converted into dummy variables. In Model 2, experience of violence was added as the primary independent variable. In Model 3, adaptive and maladaptive cognitive-emotion regulation strategies were incorporated to assess their incremental contribution to explaining PTS variance. Comprehensive diagnostic testing was conducted to verify model assumptions. The Durbin-Watson statistic was 1.800, indicating no autocorrelation issues. Multicollinearity was assessed through tolerance (all values > 0.10, range: 0.35–0.89) and variance inflation factor (VIF; all values < 10, range: 1.12–2.85), confirming no multicollinearity problems. Residual analysis verified that the model satisfied assumptions of linearity, normality of error terms, and homoscedasticity. Scatter plots of standardized residuals against predicted values showed random distribution with no systematic patterns. Cook’s distance values were all below 1.0, indicating no influential outliers. These diagnostics confirmed the appropriateness of hierarchical multiple regression for analyzing the data.

The regression model in Model 1 was statistically significant (F = 3.49, *p* = 0.002), with an explanatory power of 11.2%. Among the variables, postgraduate-level education or higher (β = −0.37, *p* = 0.008) had a significant negative effect on PTS, indicating that higher levels of education were associated with lower PTS. The regression model in Model 2, which added experiences of violence, was also statistically significant (F = 12.82, *p* < 0.001), with an explanatory power of 40.5%, an increase of 29.3% compared to Model 1. The main factors influencing PTS were experiences of violence (β = 0.63, *p* < 0.001) and graduate-level education or higher (β = −0.30, *p* = 0.008). This result confirmed that PTS increased with more experiences of violence and decreased with higher levels of education. The regression model in Model 3, which included adaptive and maladaptive emotion regulation strategies, was statistically significant (F = 24.31, *p* < 0.001), with an explanatory power of 62.6% (adjusted R^2^ = 0.626), accounting for an additional 22.1% of variance compared to Model 2 (ΔR^2^ = 0.221, *p* < 0.001). The main factors associated with PTS were maladaptive emotion regulation strategies (β = 0.56, 95% CI [0.77, 1.21], *p* < 0.001), experiences of violence (β = 0.36, 95% CI [0.30, 0.66], *p* < 0.001), and graduate-level education (β = −0.21, 95% CI [−23.59, −1.79], *p* = 0.023). The confidence intervals indicate that all significant predictors had effect estimates that were precise and excluded zero, supporting the robustness of these findings. Adaptive emotion regulation strategies had no significant direct effect on PTS (β = −0.08, *p* = 0.164). These results indicate that higher levels of education were associated with lower PTS, while more frequent experiences of violence and greater use of maladaptive emotion (Table 4).

## 4. Discussion

This study examined how psychiatric nurses’ exposure to different forms of workplace violence relates to their cognitive–emotion regulation strategies and PTS. Rather than focusing solely on violence frequency, the findings suggest that verbal aggression although often minimized or underreported in clinical practice may be associated with psychological stressor. Within psychiatric units, where emotional labor and patient unpredictability are constant, repeated exposure to verbal or threatening interactions may be correlated with higher levels of trauma-related symptoms. This interpretation highlights that even nonphysical forms of violence should be considered clinically relevant sources of psychological harm. Importantly, this study extends prior work by demonstrating that verbal and threatened forms of aggression are associated with trauma-related symptom levels that are comparable to those observed for physical violence. This provides empirical support for expanding institutional violence-prevention policies beyond incidents of physical harm.

These findings can also be interpreted through established theoretical frameworks. Conservation of Resources theory suggests that repeated exposure to verbal or threatening interactions depletes emotional resources, increasing vulnerability to trauma symptoms. Likewise, Gross’s model of emotion regulation explains why reliance on maladaptive strategies may intensify negative affect and hinder recovery after violent events. Applying these frameworks helps situate the present results within broader mechanisms of occupational stress and trauma responses.

This study found that the most common type of violence experienced by psychiatric nurses was verbal abuse. This finding is consistent with the results of several previous studies worldwide that have examined the extent of violence experienced by psychiatric nurses [26,27,28]. Despite the high frequency of verbal violence, a systematic literature review revealed a tendency to either not recognize it as a form of violence or to underreport it as insignificant [29]. However, violence of any kind is a traumatic event that threatens personal safety; therefore, institutions must be vigilant and take proactive measures. It is crucial not only to prevent violence and minimize its incidence but also to provide an immediate response and ongoing psychological and financial support to those who have experienced violence. These findings should be understood within the specific context of South Korean psychiatric healthcare settings, where hierarchical organizational structures and cultural factors may influence how violence is perceived, reported, and addressed. In Korean culture, with its emphasis on harmony and respect for authority, nurses may be less likely to report verbal violence or may normalize certain aggressive behaviors from patients. Additionally, institutional practices regarding incident reporting and support systems may differ from those in Western contexts, potentially affecting both the frequency of reported violence and the development of PTS.

Furthermore, in this study, the mean PTS score among psychiatric nurses was 37.14 out of 80, which is higher than the 31–33 point cutoff for PTS suggested in previous studies. This indicates a need for attention and intervention for PTS among psychiatric nurses. For comparison, a study using the same tool to assess emergency department nurses in the United States found a mean PCL-5 score of 14.25, with only 11% exceeding the cutoff (≥31) [30]. Similarly, in the case of novice nurses in the United Kingdom, while their mean score increased over time, it remained below the 31–33 point cutoff [31]. Additionally, a study of American adults who had experienced serious injuries and life-threatening events [32] reported a mean PCL-5 score of 21.61, while a study of female firefighters in the US [33] found a mean score of 17.07. Although direct cross-professional comparisons are challenging, these findings suggest that the level of PTS among psychiatric nurses is not negligible.

This study examined the correlation between mental health nurses’ experiences of violence, their cognitive-emotion regulation strategies, and PTS. The results revealed a positive correlation between experiences of violence and PTS, with significant positive correlations observed for verbal violence, physical threats, and physical violence. These findings are consistent with studies on psychiatric nurses [34], general nurses [13], and emergency department nurses [18]. This suggests that PTS is associated not only with direct physical violence but also with experiences of physical threats and verbal abuse. Therefore, mental health support programs should include all nurses who have experienced physical, threatened, or verbal violence. Specifically, the cumulative effect of violence from patients in the workplace and the resulting PTS can adversely impact the well-being of psychiatric nurses, along with their job satisfaction, turnover intention, quality of care, and overall job performance. Consequently, various measures must be explored to mitigate these negative effects, such as developing programs for violence prevention and response, stress management, crisis intervention, and establishing institutional mechanisms for post-incident support.

Furthermore, maladaptive emotion regulation strategies were identified as the most significant factor influencing PTS among psychiatric nurses. This finding is supported by previous research, which has also established maladaptive emotion regulation as a key factor in the development of PTS [14,35,36]. The results were consistent with earlier studies indicating that the overuse of maladaptive strategies to reduce negative emotions associated with traumatic events tends to exacerbate those very emotions [19,37,38]. At the same time, it is also possible that higher levels of PTS may lead to greater use of maladaptive regulation strategies, suggesting a potential reverse or bidirectional relationship. Conversely, adaptive emotion regulation strategies were found to have no significant effect on the nurses’ PTS. This outcome aligns with previous studies, which suggest that the intensity of anxiety and stress may hinder the use of adaptive strategies, or that the worsening of negative emotions caused by maladaptive strategies may interfere with their effective application [19,36].

In fact, the role of adaptive emotion regulation strategies is not to encourage active confrontation with problematic situations, but to aid in emotional regulation through functional methods and promote positive outcomes, such as post-traumatic growth [36]. Therefore, given that adaptive emotion regulation strategies have limitations in directly reducing PTS, educational interventions are vital. Such interventions may help individuals who have experienced violence to increase awareness of their use of maladaptive strategies and to explore ways of applying adaptive ones.

The second factor affecting PTS in psychiatric nurses was exposure to violence. This finding is consistent with the results of this study, as research focused on psychiatric nurses [5], general nurses [37], and meta-analyses [13] has shown that the frequency of exposure to violence is associated with PTS. These violent situations experienced by psychiatric nurses threaten staff safety, hinder the formation of trusting relationships with patients, and can lead to PTS. Therefore, it is necessary to explore various measures—such as developing violence prevention and response programs, stress management, crisis intervention, and post-incident support systems—to minimize violent situations and prevent them from escalating into PTS. Finally, educational attainment was found to have a significant influence on PTS, with higher educational attainment being associated with lower levels of PTS. This may be because a higher educational attainment improves self-care skills, enabling individuals to use various stress-reduction methods in stressful situations. However, further studies are needed for a more in-depth analysis. Additionally, because this study was conducted within the cultural and clinical context of Korean psychiatric nursing, the findings may reflect characteristics unique to this setting. Therefore, caution is needed when generalizing the results to other cultural or healthcare environments.

This study confirmed that maladaptive cognitive-emotion regulation strategies, experiences of violence, and educational attainment were associated with PTS in psychiatric nurses, with greater use of maladaptive strategies, more frequent violence exposure, and lower educational attainment correlated with higher PTS levels. Therefore, reducing PTS in psychiatric nurses requires, such as developing violence prevention and response programs, stress management, crisis intervention, and institutional mechanisms for post-incident support, in addition to conducting ongoing research. These initiatives should aim to minimize violent situations, assess the use of cognitive-emotional regulation strategies, and help nurses improve their self-regulation and enhance their understanding of how they use maladaptive regulation strategies. While the current study identified maladaptive cognitive-emotion regulation strategies and violence exposure as significant predictors of PTS, future research should examine whether these relationships are moderated by individual or contextual factors. Understanding such conditional effects would enable healthcare organizations to identify nurses at highest risk and develop targeted prevention strategies. For instance, if adaptive strategies are found to buffer the effects of violence exposure, interventions could prioritize strengthening these skills among nurses in high-risk settings. Similarly, identifying demographic or workplace characteristics that moderate the violence-PTS relationship would inform risk stratification and resource allocation decisions.

This study has several methodological limitations that should be considered when interpreting the findings. First, the cross-sectional design limits causal inference. While we identified associations among violence exposure, cognitive-emotion regulation strategies, and PTS, we cannot determine temporal precedence or rule out reverse causality. For example, higher PTS levels may lead to greater use of maladaptive regulation strategies, rather than the reverse. Second, the online recruitment method may have introduced self-selection bias. This study was conducted through an online forum, which may have resulted in overrepresentation of younger, technology-proficient nurses. Participants had relatively low average clinical experience (approximately 3 years), suggesting that more experienced nurses may have been underrepresented. This sampling characteristic limits the generalizability of findings across different age groups and experience levels. Additionally, differences in violence exposure or PTS severity between participants and non-participants cannot be assessed. Third, because all variables were measured using self-reported questionnaires at a single time point, common method bias may have inflated the observed associations. Although Harman’s single-factor test could not be conducted with the available dataset, this potential bias should be acknowledged. Future studies should consider incorporating objective measures or multi-informant data where feasible. Fourth, the online survey format, while offering advantages in national sampling and participant convenience, lacks direct researcher supervision. This may increase vulnerability to response bias or random responding, although our procedures (e.g., required responses for all items) helped minimize incomplete data.

## 5. Conclusions

This study was conducted to identify the effects of experiences of violence and cognitive-emotion regulation strategies on PTS in mental health nurses, with the goal of developing strategies to reduce PTS among those who have experienced violence. These findings indicate meaningful associations among violence exposure, maladaptive regulation strategies, and PTS in psychiatric nurses. Therefore, the reduction in PTS rates among psychiatric nurses may benefit from the development and implementation of violence prevention and response programs, stress and mental health management programs, and educational programs that help nurses minimize violent situations, improve self-regulation, and reduce their reliance on maladaptive cognitive regulation strategies. Such programs may include simulation-based violence-response training, trauma-informed stress-management interventions, or cognitive–emotion regulation skill-building education tailored to psychiatric nursing settings. In addition to these practical implications, the findings also offer theoretical insight by highlighting how maladaptive cognitive–emotion regulation strategies may function as important correlates of PTS within psychiatric nursing contexts.

Based on these findings, the following recommendations are proposed. First, since this study’s participants were predominantly younger and proficient with online surveys, future research should be conducted with a broader age range to improve the generalizability of the results. Second, research is needed to develop and validate the effectiveness of stress management and educational programs that focus on the use of cognitive-emotion regulation strategies. Future research would benefit from longitudinal or mixed-method designs to address the limitations of the current cross-sectional approach. Future studies may also consider incorporating more recent trauma-informed care frameworks and evidence-based workplace violence intervention models, which have been increasingly emphasized in international research. Third, follow-up studies are required to investigate other factors that may influence PTS in psychiatric nurses beyond the variables identified in this study. Fourth, we recommend conducting studies to examine the mediating or moderating effects of cognitive-emotion regulation strategies on the relationship between experiences of violence and PTS among psychiatric nurses. Fifth, we recommend conducting moderation and interaction analyses to examine the conditional nature of relationships among violence exposure, cognitive-emotion regulation strategies, and PTS. Specifically, future studies should investigate the following: (1) whether adaptive strategies buffer the negative effects of violence exposure on PTS (protective moderation), (2) whether maladaptive strategies amplify the violence-PTS relationship (risk moderation), (3) whether the effectiveness of emotion regulation strategies varies by individual characteristics such as clinical experience or educational background, and (4) whether there are synergistic effects between different types of regulation strategies. Such investigations would contribute to understanding individual differences in vulnerability and resilience, and could inform the development of personalized intervention programs tailored to nurses’ specific risk profiles and coping patterns. Sixth, we recommend that future studies expand the range of variables assessed to include potential confounders not measured in the current study. Finally, we suggest research on institutional policy effectiveness and organizational support systems.

## Figures and Tables

**Table 1:** Differences in Post-Traumatic Stress Among Psychiatric Nurses by General Characteristics (N = 140).

Characteristics	Categories	N	%	Post-Traumatic Stress				
				M	SD	t/F	*p*	Scheffe
Gender	male	10	7.1	35.30	18.80	−0.35	0.729	
	female	130	92.9	37.28	17.23			
Age (year) * 30.76 ± 3.73	20~<30	57	40.7	41.11	20.69	2.60	0.078	
	30~<35	59	42.1	34.46	12.57			
	35≤	24	17.2	34.29	17.27			
Religion	have	89	63.6	35.58	14.92	−1.41	0.161	
	have not	51	36.4	39.84	20.66			
Level of education	diploma	6	4.3	44.83	12.37	6.48	0.002	c < b < a
	bachelor’s degree	122	87.1	38.33	17.07			
	master’s degree	12	8.6	21.17	13.16			
Marital status	unmarried	111	79.3	38.40	17.22	1.70	0.091	
	married	29	20.7	32.31	16.96			
Types of hospitals	university hospital	64	45.7	34.75	14.48	3.52	0.032	a,b < c
	general hospital	35	25.0	34.57	12.39			
	psychiatric hospital	41	29.3	43.05	22.94			
Job position	staff nurse	131	93.6	37.37	17.30	0.60	0.549	
	charge nurse	9	6.4	33.78	17.61			
Certifications	have	94	67.1	36.51	15.50	−0.61	0.543	
	have not	46	32.9	38.41	20.58			
Total clinical experience (years) * 5.67 ± 3.12	<3	30	21.4	48.33	19.84	5.93	0.001	b,c,d < a
	3~<5	34	24.3	33.82	12.55			
	5~<8	49	35.0	34.51	16.66			
	8≤	27	19.3	33.63	16.14			
Psychiatric clinical experience (years) * 3.28 ± 1.83	<3	77	55.0	33.63	16.14	2.41	0.093	
	3~<5	34	24.3	39.77	17.80			
	5≤	29	20.7	35.68	13.74			
Number of emergency response training sessions	<5	106	75.7	31.86	18.71	2.96	0.055	
	5~<10	20	14.3	39.04	17.22			
	10≤	14	10.0	32.90	19.37			
Preferred plan in case of experiencing violence (duplicate response)	counseling service	89	63.6					
	change the patient in charge	94	67.1					
	emergency off	90	64.3					
	etc	3	2.0					

* M ± SD; M = Mean; SD = Standard Deviation.

**Table 2:** Levels of Violence Experience, Cognitive-Emotion Regulation Strategies, and Post-Traumatic Stress (N = 140).

Variables	Mean	SD	Min	Max	Range	Skewness	Kurtosis
Experience of violence	28.60	13.13	6	57	0–64	0.61	−0.37
verbal violence	9.01	3.43	2	16	0–16	0.04	−0.92
physical threat	9.68	4.67	2	20	0–20	0.43	−0.75
physical violence	9.91	6.70	0	25	0–28	0.66	−0.18
Adaptive emotional regulation strategy	65.77	13.29	28	95	20–100	−0.17	−0.44
Maladaptive emotional regulation strategy	47.18	9.75	18	75	16–80	0.33	0.31
Post-Traumatic Stress	37.14	17.28	0	71	0–80	0.31	−0.51

SD = Standard Deviation; Min = Minimum; Max = Maximum.

**Table 3:** Correlation between Psychiatric Nurses’ Experiences of Violence, Cognitive-Emotion Regulation Strategies, and Post-Traumatic Stress (N = 140).

Variables	1	2	3	4
	r(*p*)	r(*p*)	r(*p*)	r(*p*)
1. Experience of violence	1			
2. Adaptive emotional regulation strategy	0.30 (<0.001)	1		
3. Maladaptive emotional regulation strategy	0.48 (<0.001)	0.26 (0.002)	1	
4. Post-Traumatic Stress	0.60 (<0.001)	0.16 (0.056)	0.72 (<0.001)	1

**Table 4:** Factors Influencing Psychiatric Nurses’ Post-Traumatic Stress (N = 140).

Characteristics	Model 1					Model 2						Model 3				
	B	SE	β	t	*p*	B	SE	β	t	*p*	B	SE	β	95% CI	t	*p*
Gender * female	1.21	5.45	0.02	0.22	0.824	5.01	4.48	0.08	1.12	0.266	0.26	3.59	0.004	[−6.84, 7.36]	0.07	0.943
Marital status * married	−2.46	4.05	−0.06	−0.61	0.545	−3.80	3.32	−0.09	−1.15	0.254	−0.43	2.66	−0.01	[−5.69, 4.83]	−0.16	0.871
Level of education * bachelor’s degree	−8.81	7.00	−0.17	−1.26	0.211	−7.13	5.74	−0.14	−1.24	0.216	−5.48	4.55	−0.11	[−14.48, 3.52]	−1.21	0.230
Level of education * master’s degree	−22.49	8.34	−0.37	−2.70	0.008	−18.47	6.84	−0.30	−2.70	0.008	−12.69	5.51	−0.21	[−23.59, −1.79]	−2.30	0.023
Types of hospitals * general hospital	−1.94	3.47	−0.05	−0.56	0.577	0.41	2.85	0.01	0.14	0.885	−1.34	2.27	−0.03	[−5.83, 3.15]	−0.59	0.557
Types of hospitals * psychiatric hospital	5.92	3.39	0.16	1.75	0.083	−4.47	3.05	−0.12	−1.46	0.146	−4.16	2.44	−0.11	[−8.99, 0.67]	−1.70	0.091
Total clinical experience (years)	−0.07	0.05	−0.16	−1.61	0.111	−0.03	0.04	−0.06	−0.71	0.478	−0.06	0.03	−0.12	[−0.12, −0.00]	−1.86	0.065
Experience of violence						0.83	0.10	0.63	8.13	<0.001	0.48	0.09	0.36	[0.30, 0.66]	5.17	<0.001
Adaptive emotional regulation strategy											−0.10	0.08	−0.08	[−0.26, 0.06]	−1.40	0.164
Maladaptive emotional regulation strategy											0.99	0.11	0.56	[0.77, 1.21]	8.92	<0.001
F(*p*)	3.49 (0.002)					12.82 (<0.001)						24.31 (<0.001)				
Adjusted R^2^	0.112					0.405						0.626				
Adjusted R^2^ change						0.293						0.221				

* Total clinical experience was entered as a continuous variable (in years); all other categorical variables were dummy-coded; Reference categories: Gender = male; Marital status = unmarried; Level of education = diploma; Types of hospitals = university hospital; SE = Standard error.

## Data Availability

The raw data supporting the conclusions of this article will be made available by the authors upon reasonable request after signing a confidentiality agreement.

## Abbreviations

The following abbreviations are used in this manuscript:

PTS	Post-traumatic stress
PTSD	Post-traumatic stress disorder
